# Mass Production of Highly Active NK Cells for Cancer Immunotherapy in a GMP Conform Perfusion Bioreactor

**DOI:** 10.3389/fbioe.2019.00194

**Published:** 2019-08-13

**Authors:** Katharina Bröker, Evgeny Sinelnikov, Dirk Gustavus, Udo Schumacher, Ralf Pörtner, Hans Hoffmeister, Stefan Lüth, Werner Dammermann

**Affiliations:** ^1^Center of Internal Medicine II, Brandenburg Medical School, University Hospital Brandenburg, Brandenburg, Germany; ^2^Department of Anatomy and Experimental Morphology, University Medical Center Hamburg-Eppendorf, Hamburg, Germany; ^3^Zellwerk GmbH – HiPer-Group, Eichstädt, Germany; ^4^Institute of Bioprocess and Biosystems Engineering, Hamburg University of Technology, Hamburg, Germany; ^5^Faculty of Health Sciences, Joint Faculty of the Brandenburg University of Technology Cottbus – Senftenberg, The Brandenburg Medical School Theodor Fontane, The University of Potsdam, Potsdam, Germany

**Keywords:** natural killer cells (NK cells), cytotoxicity, tumor immunity, immunotherapy, perfusion bioreactor, GMP, mass production process

## Abstract

NK cells have emerged as promising candidates for cancer immunotherapy, especially due to their ability to fight circulating tumor cells thereby preventing metastases formation. Hence several studies have been performed to generate and expand highly cytotoxic NK cells *ex vivo*, e.g., by using specific cytokines to upregulate both their proliferation and surface expression of distinct activating receptors. Apart from an enhanced activity, application of NK cells as immunotherapeutic agent further requires sufficient cell numbers and a high purity. All these parameters depend on a variety of different factors including the starting material, additives like cytokines as well as the culture system. Here we analyzed PBMC-derived NK cells of five anonymized healthy donors expanded under specific conditions in an innovative perfusion bioreactor system with respect to their phenotype, IFNγ production, and cytotoxicity *in vitro*. Important features of the meander type bioreactors used here are a directed laminar flow of medium and control of relevant process parameters. Cells are cultivated under “steady state” conditions in perfusion mode. Our data demonstrate that expansion of CD3^+^ T cell depleted PBMCs in our standardized system generates massive amounts of highly pure (>85%) and potent anti-cancer active NK cells. These cells express a variety of important receptors driving NK cell recruitment, adhesion as well as activation. More specifically, they express the chemokine receptors CXCR3, CXCR4, and CCR7, the adhesion molecules L-selectin, LFA-1, and VLA-4, the activating receptors NKp30, NKp44, NKp46, NKG2D, DNAM1, and CD16 as well as the death ligands TRAIL and Fas-L. Moreover, the generated NK cells show a strong IFNγ expression upon cultivation with K562 tumor cells and demonstrate a high cytotoxicity toward leukemic as well as solid tumor cell lines *in vitro*. Altogether, these characteristics promise a high clinical potency of thus produced NK cells awaiting further evaluation.

## Introduction

Natural killer cells are innate effector lymphocytes which mediate potent immune responses against stressed, malignant as well as virus-infected cells. In contrast to B and T cells, they can act immediately and do not require any priming or antigen presentation. NK cells kill target cells basically via two different pathways. The exocytosis pathway involves the release of cytotoxic granules containing perforin and granzymes as well as the secretion of pro-inflammatory cytokines including IFNγ which induce target cell lysis (Smyth et al., 2005). In this context, NK cell activation depends on a balance between signals generated from different germline-encoded activating as well as inhibitory receptors. The sum of all these signals then induces either NK cell activation or inhibition. Activating receptors recognize ligands found on injured and infected cells including cells coated with IgG antibodies whereas inhibitory receptors recognize ligands found on healthy cells including MHC-I molecules. The second pathway is mediated via interaction of death receptors on the target cell and their cognate ligands on the NK cell resulting in apoptosis of the target cell (Zamai et al., 1998; Smyth et al., 2001). Target cell killing requires a tight contact between NK cell and target cell initiated predominantly by the integrin LFA-1 and its corresponding ligands. The then formed so called immunological synapse (IS) ensures the directed release of death-inducing substances and facilitates additional cell-cell interactions.

NK cells are able to infiltrate solid tumors and a high density of NK cell infiltration within the tumor mass has already been shown to be linked to a better patient prognosis (Coca et al., 1997; Gras Navarro et al., 2015; Xu et al., 2016). By controlling circulating tumor cells in the blood stream NK cells are moreover known to prevent formation of metastases (Krasnova et al., 2017) which account for ~90% of all cancer-deaths (Chaffer and Weinberg, 2011). These functions make NK cells prime candidates for cancer immunotherapy.

Several studies have been performed to assess the potential use of *ex vivo* expanded NK cells comparing autologous and allogeneic NK cells. Adoptive transfer of autologous NK cells did not have the desired success due to the inhibition of self-HLA molecules as well as limited expansion and function of the NK cells what might be explained by the, in most cases, heavy pretreatment of the patients (Geller et al., 2011). Due to these limitations, the focus of research shifted to allogeneic NK cells which turned out to be promising in clinical trials (Geller and Miller, 2011; Geller et al., 2011). Since tumor cells have evolved several different strategies to evade NK cell-mediated killing like shedding of ligands for activating receptors or upregulation of MHC-I (Pardoll, 2015), *ex vivo* manipulation of NK cell receptor expression is a promising tool to overcome immune response inhibition (Granzin et al., 2017).

Clinical application of NK cells (natural or genetically modified CAR-NK cells) (Daher and Rezvani, 2018) requires cultivation processes that aim at generating large amounts of NK cells with a high purity. The optimization of appropriate expansion processes depends on a variety of factors including the starting material, additives like cytokines as well as the culture system itself. Meanwhile, various different and extremely heterogeneous expansion processes have been described (Childs and Berg, 2013; Granzin et al., 2015; Pörtner et al., 2017). Techniques for expansion of immune cells include simple culture flasks, multi-layered flasks (such as Millicell, Millipore, or BD Multi-flask etc.), microcarrier techniques as well as special culture systems (e.g., G-Rex, WAVE-type bioreactors, Zellwerk's Z®RP Cell Breeder, Miltenyi's CliniMACS Prodigy®, the TERUMO Quantum system) (Pörtner et al., 2017, 2019). All the equipment materials are single use products which is of advantage with respect to handling and sterility.

The main drawback of most of these techniques is that they are mostly operated in batch mode. Therefore, nutrient and metabolite concentrations in the medium are continuously changing during cultivation which results in changing phenotype composition present in the harvested immune cell preparations. These inconsistent culture conditions presumably contribute to the incongruent reports of many clinical trials. To ensure reproducibility of immune cell production for cell therapies, process conditions must be controlled, evaluated, documented and validated. Continuous dynamic control of temperature, pH and pO_2_ in the medium during the immune cell expansion process is therefore indispensable as these parameters critically influence cellular behavior. Furthermore, glucose and lactate concentration as lead substances for substrates and metabolites should also be under steady control during processing. In this context, perfusion systems create a homogenous environment and allow controlled dynamic medium and gas exchange resulting in a high cell density with flexible process control.

**Figure 1:**
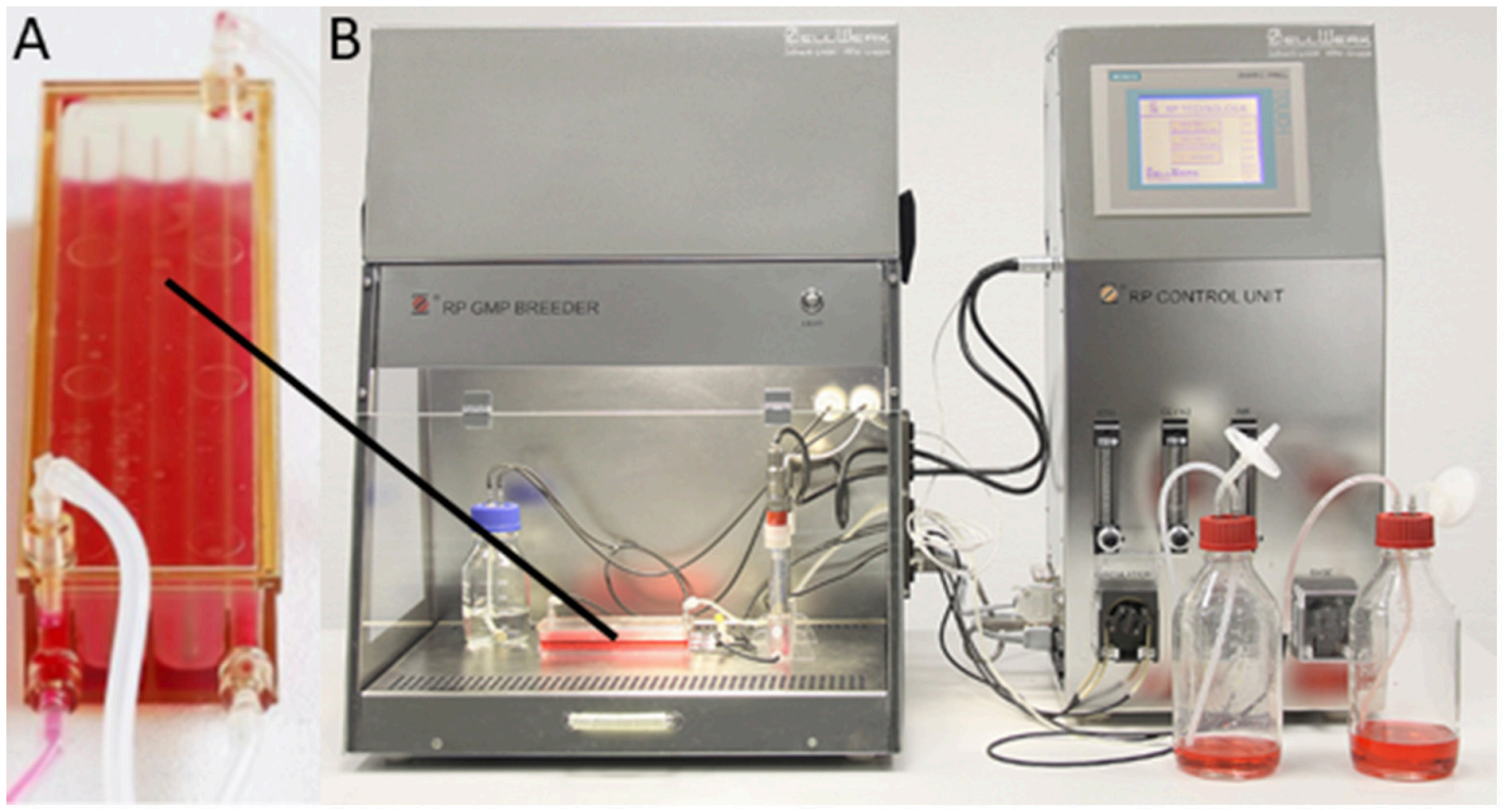
Zellwerk's Z®RP platform. **(A)** M type Bioreactor 50M; **(B)** GMP Z®RP Cell BREEDER equipped with M type Bioreactor 50M, suited for manufacturing mass amounts of NK cells/T cells/tumor infiltrating lymphocytes/mesenchymal stem cells; control unit; proprietary software.

Within the studies discussed here, an innovative perfusion process for expansion of human NK cells was developed and evaluated. The process is based on the Z®RP platform of Zellwerk and the belonging meander type bioreactors, which provide sophisticated features for mass production of different immune cells and allow cell culture and isolation within a functionally closed environment (Figure 1; Diederichs et al., 2009; Lavrentieva et al., 2013; Reichardt et al., 2013; Neumann et al., 2014; Pörtner et al., 2017). Bioreactors of the Z®RP system can be operated in the GMP Breeder. The platform automatically regulates key parameters of cultivation processes (pH, pO_2_, medium temperature, medium perfusion, feeding rate). Thus, the perfusion bioreactor process guarantees homogeneous supply of cultured cells with nutrients and gasses. Moreover, passaging of the cells is not required. Proprietary software allows automatic documentation and evaluation of the process data. The cell cultivation platform and its single use bioreactor enable the manufacturing of large quantities of individual immune cell preparations under GMP conditions.

An important feature of the meander type bioreactor used here is the directed laminar flow of medium, which allows an undisturbed cell/cell- and cell/surface-contact and minimizes cell stress (Egger et al., 2013; Neumann et al., 2014). The ratio of medium circulation and fresh medium flow is automatically regulated over time by a chosen algorithm guaranteeing a consistent homogenous supply with nutrients and gasses as well as a precise regulation of pO_2_, pH and temperature in the medium. In preliminary studies, this system has been successfully used for expansion of NK cells, T cells and tumor infiltrating lymphocytes (TILs) up to more than 10^10^ cells in a single closed cultivation run with expansion factors of 5,000–50,000 (Pörtner et al., 2017). Further optimization was dedicated to coating of seeding areas with specific matrices/antibodies to promote suppression or expansion of several immune cell species including T cell subsets and CD56^+^ NK cells (Pörtner et al., 2019).

In the present study we analyzed Z®RP expanded human NK cells that have been expanded from CD3^+^ T cell-depleted PBMCs of five different anonymized donors in an innovative perfusion production process. The focus was on the characterization of the expanded NK cells with respect to the expression levels of various functionally relevant surface molecules including chemokine receptors, cell adhesion molecules, activating receptors, the Fc receptor CD16, death ligands as well as IFNγ expression. Moreover, we analyzed the “killing capacity” of the expanded NK cells using different *in vitro* assays to evaluate their potential for cancer immunotherapy.

The main intention of our studies was the evaluation of performance and reproducibility of the newly developed process. As outlined in the following, the results underline that well controlled perfusion processes are a promising alternative to state-of-the-art-expansion techniques. As the main goal of these studies was a “proof of concept,” a deeper comparison of different culture techniques will be subject of studies in the future.

## Materials and Methods

### Antibodies and Reagents

The following antibodies were used in line with this study: anti-CD3 BV605 (OKT3), anti-CD14 BV421 (HCD14), anti-CD19 BV650 (HIB19), anti-CD45 BV570 (HI30), anti-CD56 BV510 (HCD56), anti-CD16 BV711 (3G8), anti-NKp46 PerCP-Cy5.5 (9E2), anti-NKp44 APC (P44-8); anti-NKp30 PE (P30-15), anti-NKGD2 PerCP-Cy5.5 (1D11), anti-DNAM1 APC (11A8), anti-TRAIL PE (PIK-2), anti-L-Selectin PerCP-Cy5.5 (DREG56), anti-Fas-Ligand PE (NOK-1), anti-CXCR3 PerCP-Cy5.5 (G025H7), anti-LFA-1 APC (HI111), anti-VLA-4 PE (9F10), anti-CCR7 PerCP-Cy5.5 (G043H7), anti-CXCR4 PE (12G5), anti-CD56 APC (HCD56), anti-CD107a AF488 (H4A3), anti-CD95 BV421 (DX2), anti-CD261 APC (DJR1), anti-CD262 PE (DJR2-4), anti-IFNγ BV421 (4S.B3), anti-Fas BV421 (DX2), anti-TRAIL-R1 APC (DJR1), anti-TRAIL-R2 PE (DJR-4), and Human TruStain FcX™ (Fc Receptor Blocking Solution). All were purchased from BioLegend. DPBS with 1% FBS (Thermo Fisher Scientific) and 0.09% sodium azide (Sigma-Aldrich) was used as FACS staining buffer.

Other reagents used in this study included: PBS (Biochrom), alpha medium (mod. MEM) (Biochrom), RPMI1640 medium (Thermo Fisher Scientific), McCoy's medium (Thermo Fisher Scientific), human serum (PAN-Biotech), heat-inactivated FBS (Thermo Fisher Scientific), penicillin/streptomycin (Thermo Fisher Scientific), L-glutamine (Biochrom), gentamycin (Biochrom), gentamycin sulfate (Biochrom), sodium hydrogen carbonate (Biochrom), glucose (Sigma Aldrich), human IL-2 (Proleukin, Novartis), human IL-2 (GenScript), RosetteSep™ Human CD3 Depletion Cocktail (Stemcell Technologies), Lymphoprep (Stemcell Technologies), Zombie NIR™ Fixable Viability Kit (BioLegend), Pierce™ LDH Cytotoxicity Assay Kit (Thermo Fisher Scientific), BD Cytofix/Cytoperm™ Plus (containg BD GolgiPlug™) (BD), and BD GolgiStop™ (BD).

### NK Cell Expansion

CD3^+^ T cell depleted PBMCs were isolated and *ex vivo* expanded in a proprietary cultivation process developed by Zellwerk GmbH (Diederichs et al., 2009; Reichardt et al., 2013; Pörtner et al., 2017). Briefly, anonymized blood products for research purposes (whole blood) from five donors were obtained, mixed with RosetteSep™ Human CD3 Depletion Cocktail and incubated for 30 min. Subsequently, the PBMC fraction was isolated using Lymphoprep in a density gradient centrifugation. The obtained PBMCs were washed twice with PBS and then directly inoculated into the bioreactor as described in the following section.

**Figure 2:**
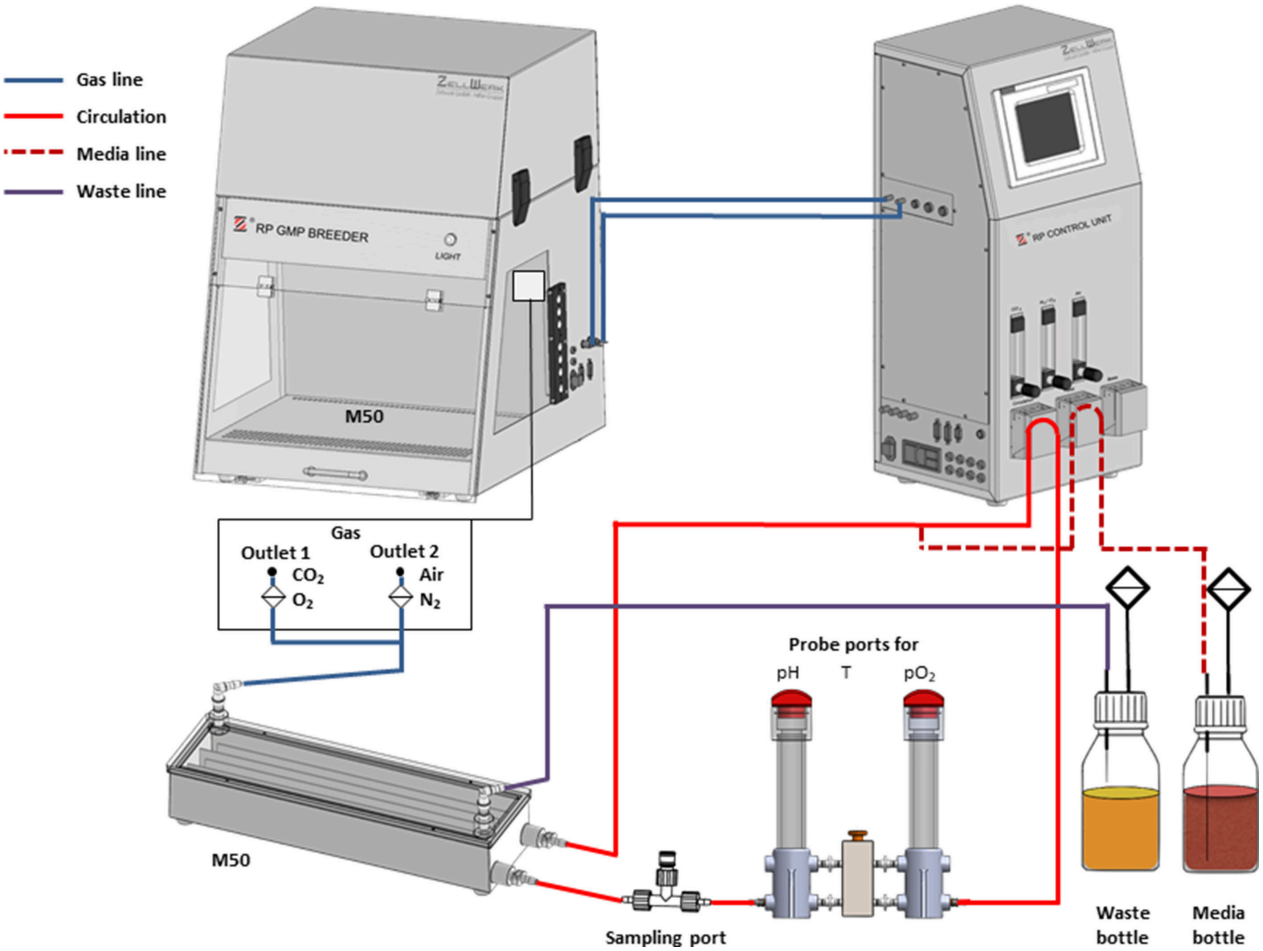
Flow chart of the bioreactor set-up. The M50 meander type bioreactor is placed in the GMP breeder during operation (compare Figure 1) and connected to gas supply. Medium is circulated in a loop by peristaltic pumps from the reactor to the sensors (pH, T, pO_2_) and from there back to the reactor. This enables inline/online analysis of the process parameters which are compared with the set points by means of the software of the Z®RP control unit. If one of the actual values for pH, pO_2_, and/or temperature is outside the tolerance range, corrective measures are undertaken for control. These include increasing the volume flow of CO_2_, O_2_, N_2_, or air and increasing the temperature of the breeder. Fresh culture medium is fed from the medium bottle into the culture system via the circulation. An overflow attached to the upper lid of the bioreactor allows the removal of excess medium and metabolites from the system into the waste bottle.

In line with our study, we used the Z®RP Bioreactor 50M operated in the cultivation platform Z®RP GMP Breeder (both from Zellwerk GmbH) (compare introduction) (Pörtner et al., 2019). As shown in Figure 2, the bioreactor itself is integrated in a circulation circuit. Prior to use, the perfusion bioreactor 50M was coated with a specific proprietary activating cocktail. NK cells growth is generally enhanced under these conditions, cell division persists for an extended period of time and harvested NK cells show much higher cytotoxicity compared to conventionally cultured NK cells (own observations, data not shown). After coating, the reactor was initially filled with 35 ml of alpha medium supplemented with glucose (2.5 g/L), 10% human serum, L-glutamine, gentamycin, 1,000 IU/ml human IL-2 (Proleukin, Novartis) as well as a proprietary activation cocktail. The bioreactor was inoculated with ~70 × 10^6^ cells. For this purpose, the cells were resuspended in 9.5 ml of medium and injected via a syringe through the sampling port into the first two rows of the bioreactor. After 2 h, when the cells had settled, medium circulation and aeration were switched on.

**Figure 3:**
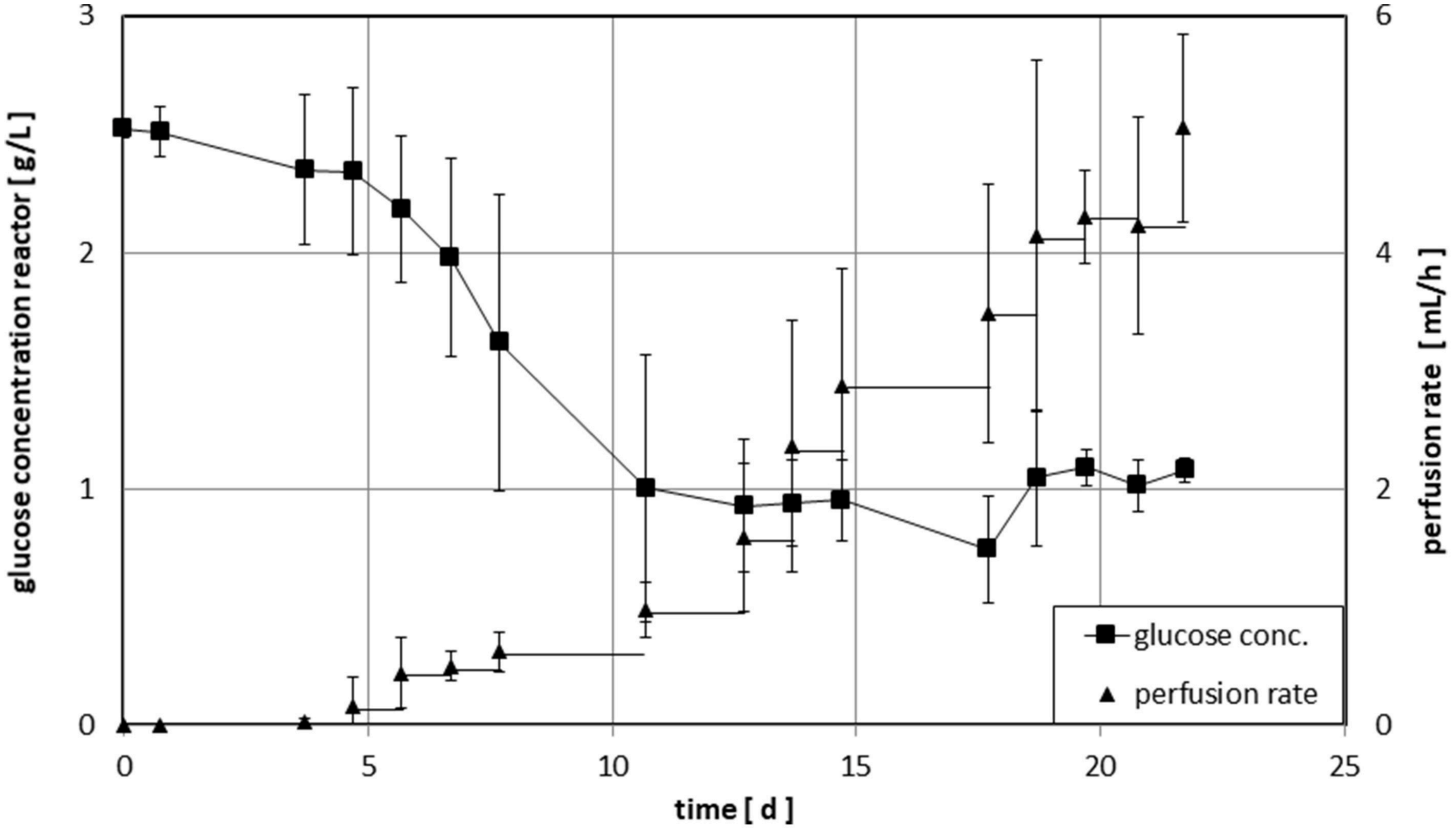
Time course of glucose concentration and perfusion rate for cultivation of NK-cells in the Z®RP Bioreactor 50M. During the first stage the bioreactor was operated without inflow of medium to let the cells start to grow. When glucose concentration reached a target value of ~1 g/L, inflow of fresh medium (perfusion rate) was adjusted manually to maintain this value. The individual runs were stopped, when a final cell number of ~1 × 10^9^ cells was reached.

The process parameters the bioreactor was operated with are listed in Table 1. During culture, 0.5 ml of medium was withdrawn via the sampling port each working day by means of a syringe. The content of lactate (data not shown) and glucose (compare Figure 3) within these samples was determined by means of a biochemical analyzer (YSI 2000 Select, YSI). During the first stage, the bioreactor was operated without inflow of medium to let the cells start to grow whereas the decrease in glucose concentration was used as an indicator for cell growth. When the glucose concentration reached a target value of ~1 g/L, inflow of fresh medium (perfusion rate) was adjusted manually to maintain this value. The cells were expanded until a final cell number of ~1 × 10^9^ was obtained. The bioreactor was shaken to suspend the cells before taking samples for cell counting. The desired cell number was achieved within a period of 12–22 days. Cells were harvested and then shipped on dry ice to the University Medical Center Hamburg-Eppendorf and stored there in liquid nitrogen until used for analytics. The number of PBMCs used as starting material and the length of cultivation are listed in Table 2.

**Table 1:** Process parameters for operation of the bioreactor.

**Parameter**	**Value**	**Specification**
Temperature	36.8°C	
pO_2_ air saturation	Flow rate = 10 mL/min	Adjustment of the oxygen content in the air flow
pH	7.3	Adjustment of the CO_2_-concentration in the air flow (start: 5%)
Circulation	1.5 mL/min	

*For NK cell expansion the bioreactor was operated with a distinct temperature, pO_2_-air saturation level, pH value, and circulation*.

**Table 2:** Donor-specific information and information concerning cell expansion.

**Donor**	**Gender**	**Age (years)**	**Number of PBMCs (× 10^**6**^)**	**Length of cultivation (days)**
1	n.d.	n.d.	148	12
2	m	29	60	22
3	m	37	70	22
4	m	56	43	16
5	m	56	59	14

*Gender and age of the blood donors, the number of PBMCs used for NK cell expansion, and the length of cultivation in the bioreactor*.

### Cell Culture

Prior to any analytical measurements, cryoconserved NK cells were pre-activated with IL-2 (GenScript). In brief, cells were thawed and immediately washed (350 g, 5 min) with alpha medium supplemented with 10% human serum, 2 mM L-glutamine, 0.05 mg/ml gentamycin sulfate, 3.1 g/l sodium hydrogen carbonate, and 2.9 g/l glucose. Cells were then re-suspended in complete alpha medium with IL-2 (100 IU/ml) and transferred into a 24-well plate (1 × 10^6^ cells/ml). The cells were incubated for 48 h at 37°C, 5% CO_2_. Afterwards, cells were collected, centrifuged (350 g, 5 min) and re-suspended in RPMI1640 medium supplemented with 5% heat-inactivated FBS, 100 U/ml penicillin and 100 μg/ml streptomycin. The cells were then pipetted on a cell strainer (40 μm nylon, BD) to remove cell debris.

The human tumor cell lines K562 (leukemia) and PaCa5061 (pancreatic cancer) were maintained in RPMI1640 medium and the human tumor cell line SKOV3 (ovarian cancer) in McCoy's medium. Both media were supplemented with 10% heat-inactivated FBS, 100 U/ml penicillin, and 100 μg/ml streptomycin.

### Phenotypic Characterization by Flow Cytometry

Phenotypic characterization of the cells was performed using a LSR Fortessa cytometer (BD). Flow cytometric analysis of the NK cells was performed to assess NK-cell purity using different lineage markers as well as to investigate the expression of chemokine receptors, adhesion molecules, activating receptors, the Fc receptor CD16 and death ligands. NK cells were analyzed both, directly after thawing as well as after IL-2 re-activation.

For live/dead discrimination NK cells were first re-suspended in DPBS (1 × 10^7^/ml) and stained with Zombie NIR™ dye for 20 min at RT. Cells were then washed with staining buffer (350 g, 5 min) and incubated in staining buffer with Human TruStain FcX™ for 10 min at RT. Subsequently, cells were stained with different sets of antibodies. Anti-CD3 BV605, anti-CD14 BV421, anti-CD19 BV650, anti-CD45 BV570, anti-CD56 BV510, anti-CD16 BV711 were used as linage markers and combined with either anti-CD335 PerCP-Cy5.5, anti-CD336 APC and anti-CD337 PE, anti-CD314 PerCP-Cy5.5, anti-CD226 APC and anti-CD253 PE, anti-CD62L PerCP-Cy5.5 and anti-CD178 PE, anti-CD183 PerCP-Cy5.5, anti-CD11a APC and anti-CD49d PE, or anti-CD197 PerCP-Cy5.5 and anti-CD184 PE antibodies. Cells were stained for 30 min at RT, washed and finally re-suspended in fresh staining buffer.

Further, all tumor cells used in this study were analyzed with regard to expression of the death receptors Fas, TRAIL-R1, and TRAIL-R2. For cell detachment adherent tumor cells (PaCa5061, SKOV3) were washed with DPBS and incubated in trypsin EDTA for 5 min at 37°C. All cells including the non-adherent K562 cells were washed with DPBS, re-suspended in staining buffer (0.5 × 10^6^/ml) and incubated with Human TruStain FcX™ for 10 min at RT. Cells were then stained with Fas BV421, TRAIL-R1 APC, and TRAIL-R2 PE for 30 min at RT, respectively. Finally, cells were washed and re-suspended in fresh staining buffer.

### LDH Cytotoxicity Assay

The cytotoxic capacity of the NK cells was assessed using a LDH cytotoxicity assay following the manufacturer's protocol. Briefly, NK cells and tumor cells were transferred in 96-well (U bottom) plates using different E:T ratios (10:1, 5:1, 2,5:1, 1:1) and incubated for 4 h at 37°C, 5% CO_2_. Three Controls were included: an effector cell spontaneous LDH release control, a target cell spontaneous LDH release control and a target cell maximum LDH release control. Supernatants were then transferred into a new plate and mixed with reaction mixture. After 30 min of incubation at RT stop solution was added. LDH activity was subsequently determined using a plate-reading spectrophotometer (Dynex Technologies). LDH release was calculated as follows:

$$\begin{array}{l} \%\;cytotoxicity=\\ \frac{\text{cell}-\text{treated LDH activity}-\text{spontaneous LDH activity}}{\text{maximum LDH}-\text{activity}-\text{spontaneous LDH activity}} \end{array}$$

Since different cell types show different levels of LDH activity, in a first step the optimum cell number of the different tumor cell lines for the LDH assay was determined to ensure that the LDH signal is within the linear range. The following cell numbers were finally used: 2 × 10^4^ K562 cells, 0.5 × 10^4^ PaCa5061 cells and 10 × 10^4^ SKOV3 cells.

### Multi-response Assay

The multi-response assay was performed using a LSR Fortessa cytometer (BD). To assess the degranulation potential of the NK cells as well as intracellular expression of IFNγ, the cells were pre-activated with IL-2 as described above. Then, they were centrifuged (350 g, 5 min), re-suspended in fresh RPMI1640 medium (5% FBS, 1% penicillin/streptomycin) and a total of 1 × 10^5^ cells (1 × 10^6^ cell/ml) was transferred into a 96-well-plate (U bottom). Subsequently, K562 cells were centrifuged (350 g, 5 min), re-suspended in RPMI1640 medium (5% FBS, 1% P/S), and a total of 2 ×10^5^ cells (2 ×10^6^ cell/ml) was added to the NK cells. Cells were incubated for 1 h at 37°C, 5% CO_2_ before 20 μL of culture medium supplemented with GolgiPlug diluted 1:100 and GolgiStop diluted 1:150 was added. Cells were incubated for 5 more hours and were then recollected, centrifuged (350 g, 5 min) and incubated in staining buffer with Human TruStain FcX™ for 10 min at RT. Subsequently, surface staining with anti-CD56-APC and anti-CD107a-AF488 antibodies was performed for 30 min at RT. The cells were then washed with staining buffer (350 g, 5 min) and incubated in fixation/permeabilization solution (Cytofix/Cytoperm) over night at 4° C. The next day, the cells were washed with perm/wash buffer (1X) (350 g, 5 min) and stained with anti-IFNγ-BV421 antibodies in perm/wash buffer for 30 min at RT. Cells were subsequently washed with staining buffer (350 g, 5 min) and re-suspended in fresh staining buffer afterwards. They were stored at 4° C and were analyzed within 1 week using flow cytometry.

### Statistical Analyses

Statistical analysis was performed using Graph Pad Prism 5 (GraphPad Software, Inc., USA). To assess differences between multiple groups, non-parametric one-way ANOVA on ranks (Kruskal–Wallis) test was used with Dunn's *post-hoc* evaluation (Figure 11).

## Results

### Perfusion Culture

The time course of the perfusion cultures within the Z®RP Bioreactor 50M is depicted in Figure 3. On average, glucose concentration dropped to the desired level of 1 g/L within ~10 days. Afterwards, the target concentration was maintained within a range of ± 20% (mean value of standard deviation after day 10 shown in Figure 3). The exponential increase of the cell density is reflected by the corresponding increase of the perfusion rate. The large standard deviation accounts for the large difference in initial cell growth of the different donors.

### NK Cell Purity

**Figure 4:**
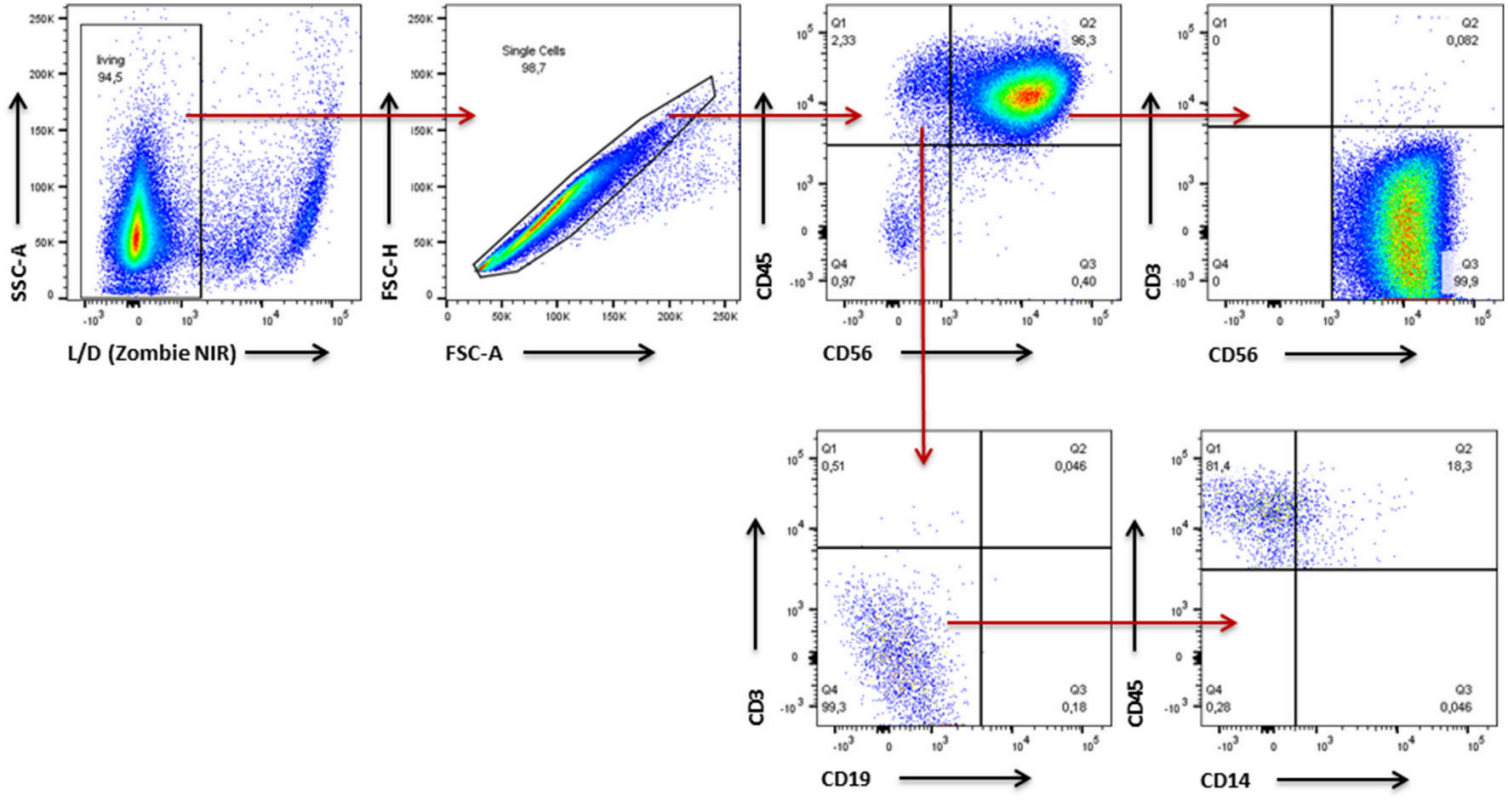
Gating Strategy for analysis of NK cell purity and expression pattern of surface molecules. Gating strategy used for flow cytometric analysis of expanded NK cells assessing NK cell purity as well as expression of surface molecules on NK cells. First, dead cells and cell doublets were excluded and CD45^+^ leukocytes were identified. Within the leukocyte population CD56^+^ NK/NK-T cells and non-NK cells were discriminated. Within the non-NK cell fraction expression of CD3 and CD19 reflected presence of T lymphocytes and B lymphocytes, respectively. Cells which were negative for CD3 and CD19 were further analyzed for CD14 expression to identify monocytes. With regard to the NK/NK-T cell fraction CD3 was used to differentiate CD56^+^CD3^+^ NK-T cells from CD56^+^CD3^−^ NK-T cells.

**Figure 5:**
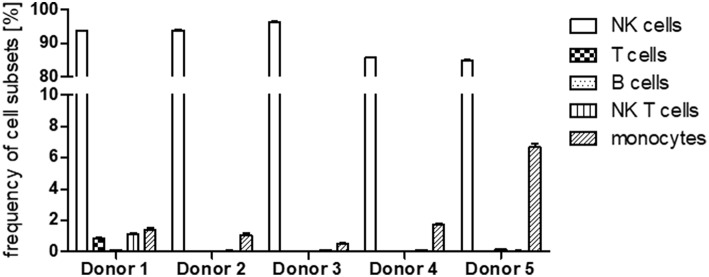
Frequency of cell subsets after expansion. Frequency of NK cells, T cells, B cells, NK T cells, and monocytes within the cells of the five different donors after cell expansion. Values shown are the mean ± SEM.

Since PBMCs served as source for the NK cells we first assessed NK cell purity in samples of all five donors using flow cytometry (Figure 4). In an initial step we excluded dead cells and cell doublets and then identified CD45^+^ leukocytes. Within the leukocyte population we discriminated CD56^+^ NK/NK-T cells and non-NK cells. Having a closer look at the non-NK cell fraction expression of CD3 and CD19 reflected presence of T lymphocytes and B lymphocytes, respectively. Cells which were negative for CD3 and CD19 were further analyzed for CD14 expression to identify monocytes. With regard to the NK/NK-T cell fraction CD3 expression was used to differentiate CD56^+^CD3^+^ NK-T cells from CD56^+^CD3^−^ NK cells. In all cases NK cell purity reached more than 85% whereas cells from donors 1–3 showed with more than 90% a higher purity than donors 4–5. The frequencies of T cells, B cells as well as NK T cells were below 2% in cells from all donors. Monocytes represented the second largest of all analyzed cell fractions after NK cells, e.g., reaching more than 6% of total cells in donor 5 (Figure 5).

### Expression of Chemokine Receptors

NK cell tissue distribution under non-inflammatory conditions as well as their recruitment to sites of inflammation and tumors is regulated by a variety of different chemokines. Thus, NK cells express various chemokine receptors on their surface (Inngjerdingen et al., 2001; Bernardini et al., 2016).

**Figure 6:**
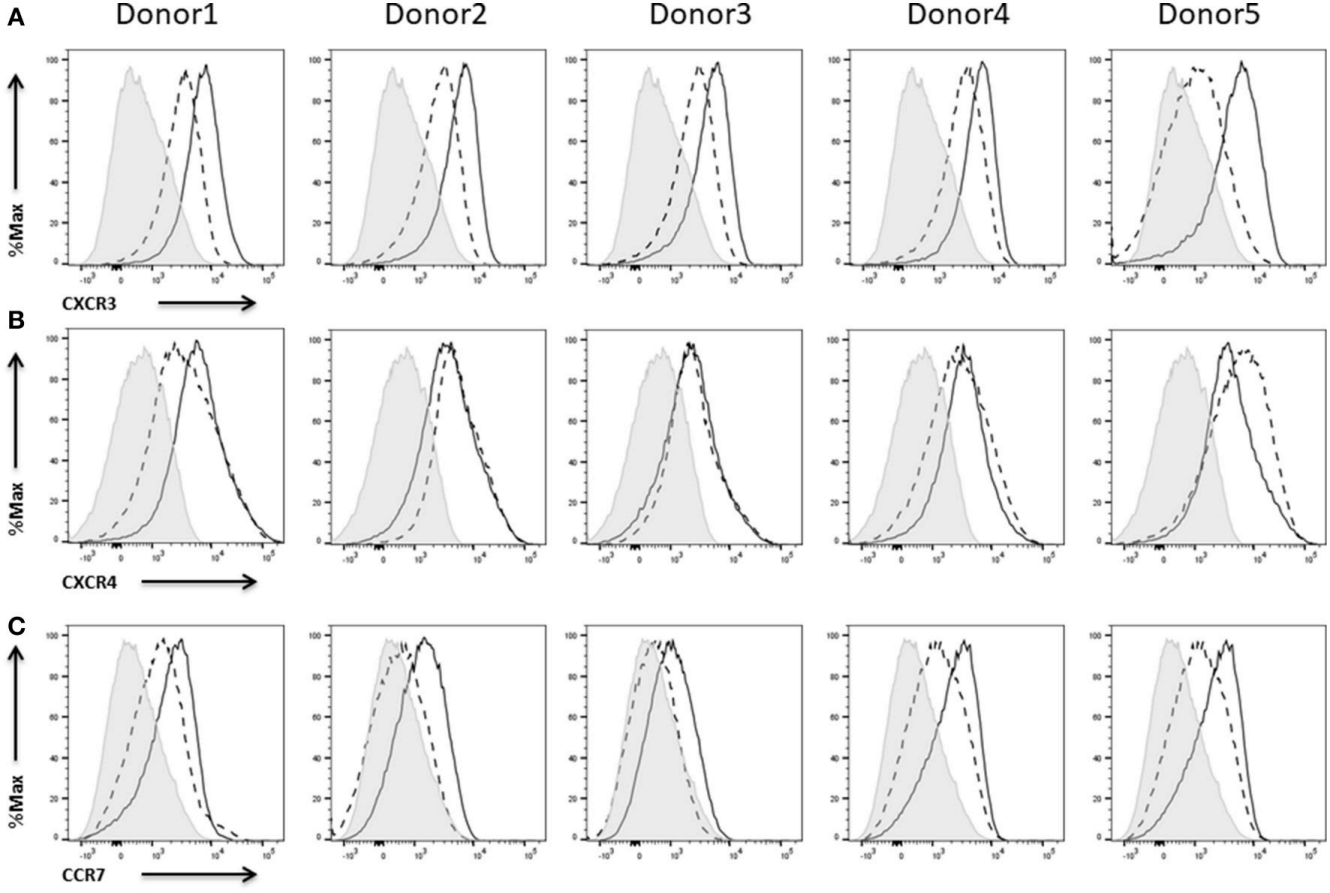
Expression of chemokine receptors on NK cells. Flow cytometric analysis of the surface expression of the chemokine receptors **(A)** CXCR3 and **(B)** CXCR4, **(C)** CCR7 on CD45^+^CD56^+^CD3^−^ NK cells of five different donors. Dashed line depicts the signal for IL-2 expanded NK cells, solid line for IL-2 re-stimulated NK cells and shaded graphs show FMO controls.

The chemokines CXCL9 and CXCL10 and their receptor CXCR3 are of paramount importance for NK cell efficacy since they drive their recruitment into solid tumors (Wendel et al., 2008; Bernardini et al., 2016). A high tumor infiltration with lymphocytes has been shown to correlate with a prolonged survival in a variety of different cancers (Kondo et al., 2004; Denkert et al., 2010). CXCR3 expression could be measured on IL-2 expanded NK cells and to a higher extend on IL-2 re-stimulated cells (Figure 6A).

The CXCR4/CXCL12 axis has been described to regulate the localization of NK cells in the bone marrow (Bernardini et al., 2008) and seems to be of importance with respect to recruitment of NK cells to the tumor site in neuroblastoma and multiple myeloma (Hanna et al., 2003; Ponzetta et al., 2015). We could already measure CXCR4 expression on freshly thawed, IL-2 expanded NK cells. Activation of the NK cells with IL-2 only had minor effects on the expression of the receptor (Figure 6B).

CCR7 mediates homing to lymphoid tissues like peripheral lymph nodes (Pesce et al., 2016). Thus, expression of this receptor on NK cells is seen as advantage with respect to targeting lymph node metastases (Somanchi et al., 2012). NK cells from three of five donors showed a rather weak CCR7 expression on IL-2 expanded NK cells whereas IL-2 re-stimulation induced or enhanced expression of the receptor (Figure 6C).

### Expression of Cell Adhesion Molecules

Like other leukocytes, NK cells require different adhesion molecules to leave the blood stream and enter peripheral tissue. Selectins binding carbohydrates are involved in initial interactions between leukocytes and endothelium resulting in rolling of the leukocytes on the endothelium. Of these selectins, only L-selectin is expressed on the leukocyte surface whereas E- and P-selectin are present on activated endothelial cells. L-selectin is moreover critical for lymphocyte homing to peripheral lymph nodes (Gallatin et al., 1983; Arbonés et al., 1994).

Strong leukocyte adhesion to the endothelium depends on integrins, basically LFA-1 and VLA-4. In their high-affinity state these integrins bind their ligands ICAM-1 and VCAM-1, respectively, resulting in firm attachment of the leukocytes to the endothelium. ICAM-1 and L-selectin have even been shown to contribute co-operatively to the anti-tumor reaction by regulating tumor lymphocyte infiltration (Yamada et al., 2006). Apart from that, LFA-1 mediates NK cell adhesion to target cells, maturation of the immunological synapse and is crucial for granule polarization and therefore efficient killing (Bryceson et al., 2005; Hoffmann et al., 2011).

**Figure 7:**
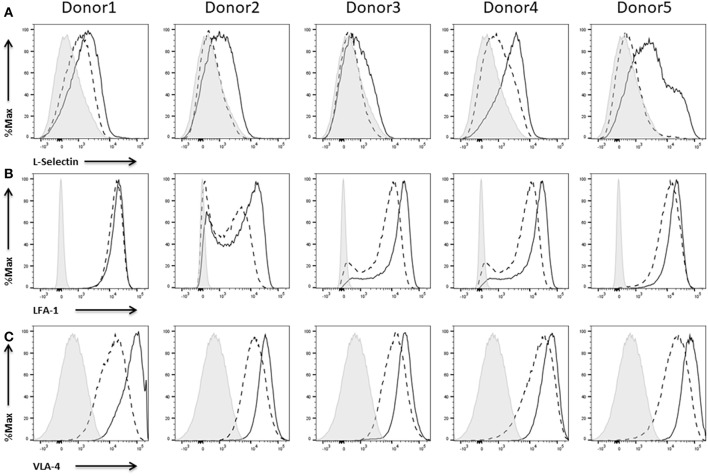
Expression of cell adhesion molecules on NK cells. Flow cytometric analysis of the surface expression of the cell adhesion molecules **(A)** L-Selectin, **(B)** LFA-1, and **(C)** VLA-4 on CD45^+^CD56^+^CD3^−^ NK cells of five different donors. Dashed line depicts the signal for IL-2 expanded NK cells, solid line for IL-2 re-stimulated NK cells, and shaded graphs show FMO controls.

According to our data, IL-2 expanded NK cells don't show any or just a low expression of L-selectin. Anyhow, upon activation with IL-2, NK cells of all donors upregulate L-selectin expression (Figure 7A). In contrast to this up-regulation, the integrins LFA-1 (Figure 7B) and VLA-4 are already expressed to a high extend on IL-2 expanded NK cells (Figure 7C). Nevertheless, IL-2 re-stimulation led to an additional increase of LFA-1 and VLA-4 expression. Only in case of donor 1 there was no increase with respect to LFA-1 expression induced by IL-2.

### Expression of Activating Receptors

NK cells are equipped with a set of different receptors which recognize molecules on the surface of other cells generating either inhibitory or activating signals. The NK cell response is in the end regulated by a balance between both signal types.

In general, activating receptors recognize molecules on injured, infected, and malignant cells. NKp30, NKp44, and NKp46 belong to the group of natural cytotoxicity receptors (NCRs) which are important mediators of NK cell cytotoxicity and some studies even suggest that the NCRs represent one of the main mechanisms by which NK cells kill tumor cell targets (Pegram et al., 2011). Thus, blocking these receptors leads to impaired killing of malignant as well as infected cells *in vitro* (Moretta et al., 2001)and low expression levels *in vivo* correlate for instance with resistance of leukemia cells to NK cell cytotoxicity in patients with acute myeloid leukemia (Costello et al., 2002; Arnon et al., 2004; Fauriat et al., 2007). Even deletion of just a single NCR has been shown to impair NK cell mediated lysis of tumor cells *in vivo* (Pegram et al., 2011).

**Figure 8:**
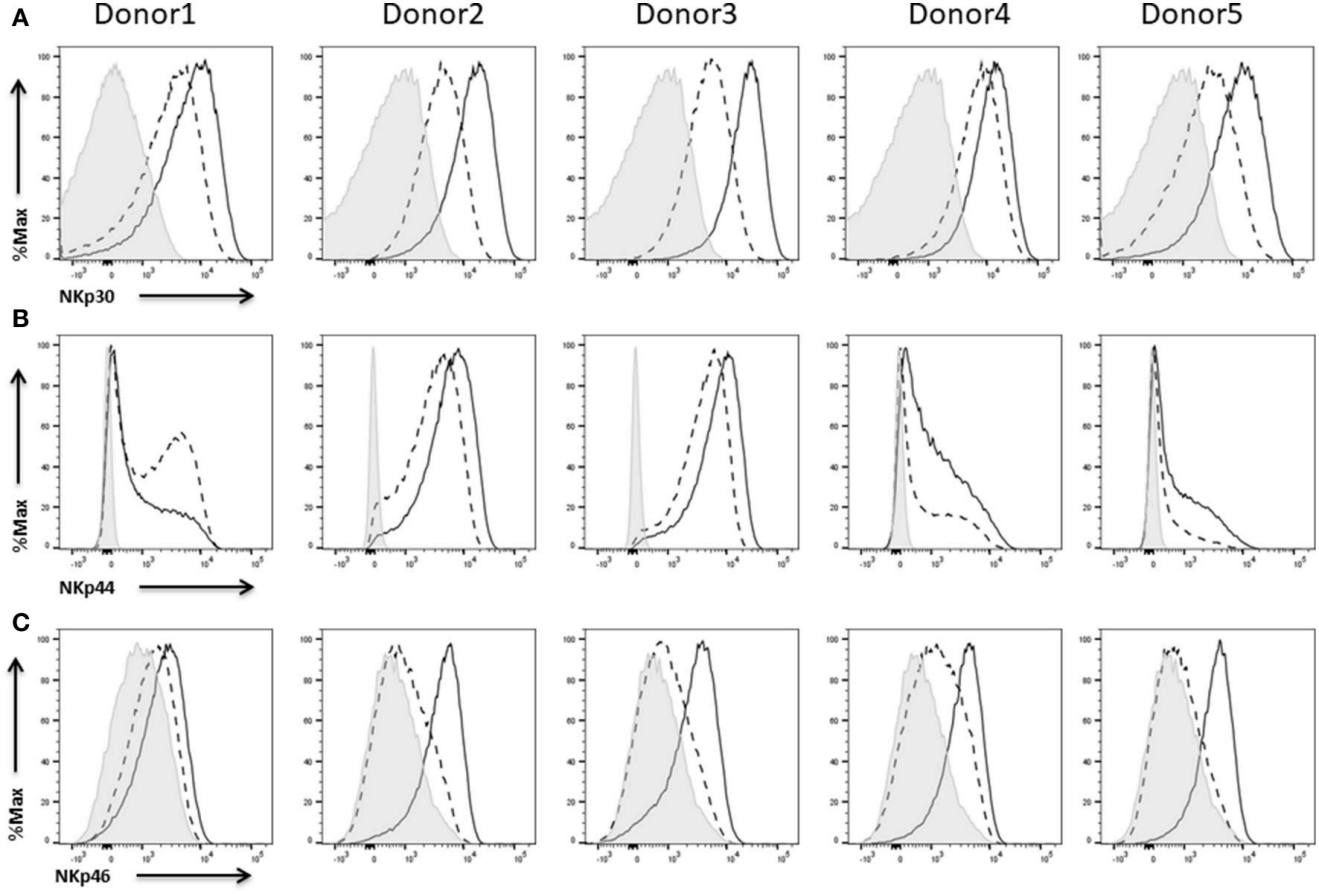
Expression of natural cytotoxicity receptors on NK cells. Flow cytometric analysis of the surface expression of the activating receptors NKp30 **(A)**, NKp44 **(B)**, and NKp46 **(C)** on CD45^+^CD56^+^CD3^−^ NK cells of five different donors. Dashed line depicts the signal for IL-2 expanded NK cells, solid line for IL-2 re-stimulated NK cells, and shaded graphs show FMO controls.

We found expression of all three receptors on the NK cells of the five different donors. NKp30 (Figure 8A) and NKp44 (Figure 8B) were already expressed on IL-2 expanded NK cells whereas NKp46 (Figure 8C) was barely present under these conditions. Anyhow, re-stimulation with IL-2 led to an increase in the expression of all three receptors. Only in case of donor 1 we could not detect any enhanced expression of NKp44 upon IL-2 activation (Figure 8B, left).

Apart from the NCRs, there are other activating receptors which are important for proper NK cell functionality including NKG2D and DNAM-1. NKG2D is crucial for surveillance toward spontaneous malignancies (Molfetta et al., 2017) and plays an important role in NK cell-mediated recognition and control of different cancer entities including B cell lymphoma, multiple myeloma, osteosarcoma, prostate cancer, cervical cancer, and childhood AML (Garcia-Iglesias et al., 2009; Belting et al., 2015; Fernández et al., 2015; Fionda et al., 2015; Pasero et al., 2015; Schlegel et al., 2015).

DNAM-1 is another important activating receptor on NK cells controlling cytotoxicity as well as IFNγ production against a wide range of transformed as well as infected cells (de Andrade et al., 2014). As NKG2D, DNAM-1 has been shown to be involved in immune responses against different tumors like prostate cancer, ovarian neoplasia and multiple myeloma (da Silva et al., 2014; Fionda et al., 2015; Pasero et al., 2015).

**Figure 9:**
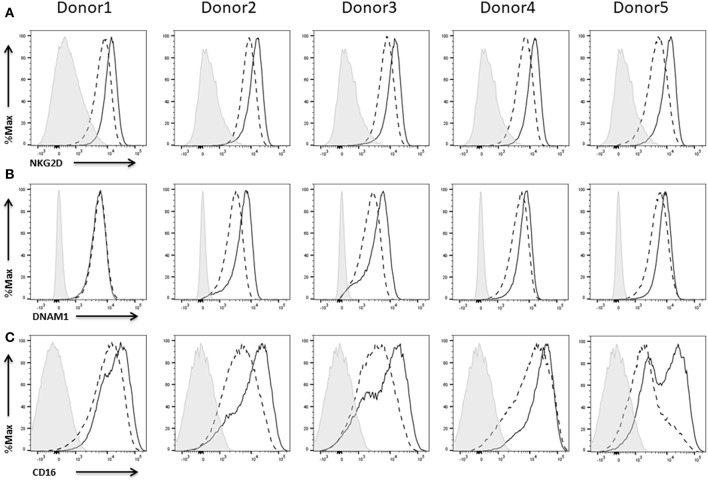
Expression of activating receptors on NK cells. Flow cytometric analysis of the surface expression of the activating receptors NKGD2 **(A)** and DNAM1 **(B)** CD16 **(C)** on CD45^+^CD56^+^CD3^−^ NK cells of five different donors. Dashed line depicts the signal for IL-2 expanded NK cells, solid line for IL-2 re-stimulated NK cells, and shaded graphs show FMO controls.

We could show strong NKG2D (Figure 9A) as well as DNAM1 (Figure 9B) expression on NK cells of all five donors. Expression of both receptors did not require IL-2 re-stimulation of the cells, but IL-2 activation enhanced the NKG2D expression levels on NK cells from all donors (Figure 9A) and DNAM1 expression levels on NK cells from donors 2–5 (Figure 9B).

As low-affinity receptor for IgG antibodies CD16 serves as another important activating receptor on NK cells promoting antibody-dependent cell-mediated cytotoxicity (ADCC).

There are different NK cell subsets described which vary with respect to phenotype and function (Cooper et al., 2001). The two main NK cell subsets have been identified according to cell surface density of CD56 and expression of CD16. In this context, the expression of CD16 is linked to the cytotoxicity as well as the cytokine production of the NK cells. Thus, CD16^+^ NK cells can spontaneously lyse targeted tumor cells whereas CD16^−^ NK cells are potent cytokine producers with only little ability to kill tumor cell targets (Caligiuri, 2008; Narni-Mancinelli et al., 2013). The majority of the NK cells of all five donors were positive for CD16. Furthermore, re-stimulation with IL-2 for 48 h led to an upregulation of CD16 expression in all cases (Figure 9C).

### Expression of Death Ligands

One of the two major mechanisms by which NK cells kill their targets involves the activation of death receptors on the target cells by binding their cognate ligands on the NK cells thereby inducing target cell apoptosis. The importance of this pathway has been emphasized by studies showing that inhibition of death receptor-mediated apoptosis goes along with enhanced tumor progression (Screpanti et al., 2001). NK cells have been shown to express the death ligands FasL and TRAIL (Zamai et al., 1998).

**Figure 10:**
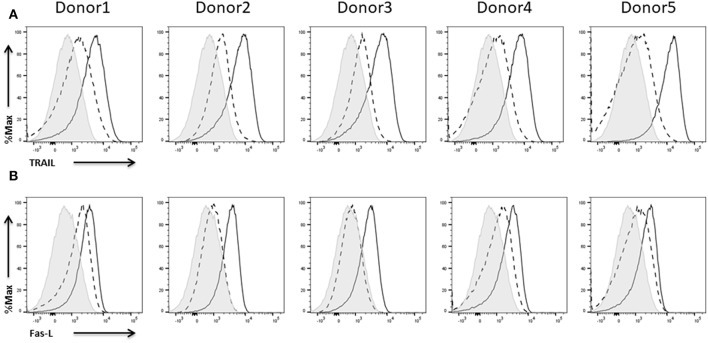
Expression of death ligands on NK cells. Flow cytometric analysis of the surface expression of the death ligands **(A)** TRAIL and **(B)** Fas-L on CD45^+^CD56^+^CD3^−^ NK cells of five different donors. Dashed line depicts the signal for IL-2 expanded NK cells, solid line for IL-2 re-stimulated NK cells, and shaded graphs show FMO controls.

The expression of TRAIL on NK cells of all five donors was rather weak before and much stronger after activation with IL-2 (Figure 10A). The results for FasL expression were quite similar. Depending on the donor, IL-2 expanded NK cells did not show any or only a low expression of FasL (Figure 10B). Anyhow, upon re-stimulation with IL-2, NK cells of all donors showed FasL expression. To better evaluate the susceptibility of the different tumor cell lines to death receptor-induced killing we analyzed death receptor expression on all three cell lines (Supplementary Figure 1).

### Cytotoxicity

**Figure 11:**
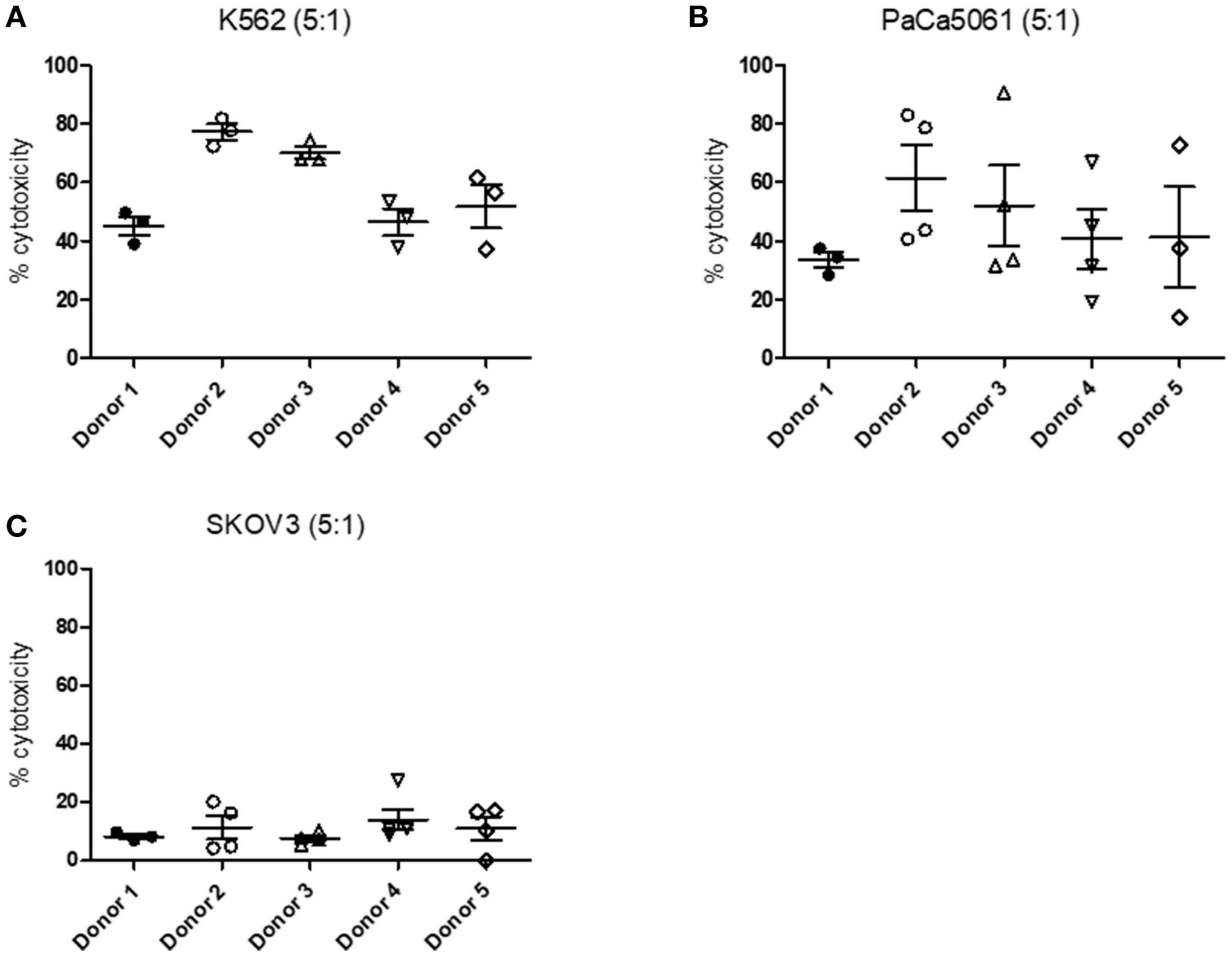
Cytotoxicity of NK cells toward different cancer cell lines measured by LDH cytotoxicity assay. Cytotoxicity measured by LDH cytotoxicity assay after cultivation of NK cells with **(A)** K562, **(B)** PaCa5061, and **(C)** SKOV3 cells. Cells were cultured with an E:T ratio of 5:1. *n* ≥ 3. Values shown are the mean ± SEM. Statistical differences between the different donors were determined using a non-parametric one-way ANOVA on ranks (Kruskal–Wallis) test with Dunn's *post-hoc* evaluation.

In order to analyze NK cell cytotoxicity toward different tumor cell lines we performed LDH assays. NK cells were therefore co-cultivated with K562, PaCa5061, and SKOV3 tumor cells, respectively. The measured cytotoxicity was dependent on both, the donor and the cell line used. The cytotoxicity toward K562 cells ranged from ~40 to 80% with NK cells from donor 2 and donor 3 showing a higher cytotoxicity than NK cells from the three remaining donors (Figure 11A). We could further measure cytotoxicity toward PaCa5061 cells which was on average between 30 and 60% (Figure 11B). Unfortunately, we observed a very high variability between single measurements. In comparison to K562 and PaCa5061 cells, SKOV3 cells were less susceptible to NK cell-mediated killing (Figure 11C). On average, cytotoxicity did not exceed 20%. In general, the measured cytotoxicity was further dependent on the E:T ratio (Supplementary Figure 2).

### Degranulation and IFNγ Expression

As an additional readout for the cytotoxic potential we assessed the degranulation of the NK cells by analyzing the expression of CD107a as degranulation marker after 6 h of cultivation in absence or presence of K562 cells using flow cytometry. In line with the same experiment, we also measured the intracellular expression of IFNγ. Briefly, after exclusion of cell doublets, NK cells were identified as CD56^+^ cells. NK cells were subsequently analyzed with respect to CD107a and IFNγ expression.

**Figure 12:**
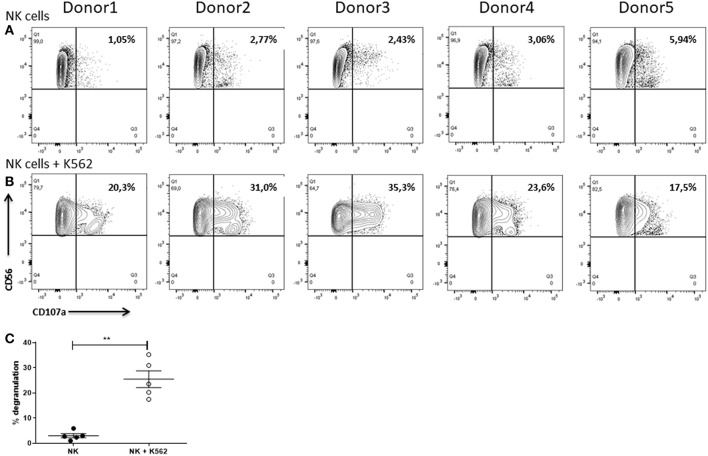
Degranulation of NK cells. Flow cytometric analysis of the surface expression of the degranulation marker CD107a on IL-2 re-stimulated NK cells after cultivation for 6 h in **(A)** absence or **(B)** presence of K562 cells. Gates were set according to FMO controls. Graphs are representative for two independent experiments. **(C)** Values shown are the mean ± SEM. Statistical differences between both groups were determined using a paired *t*-test. *p = 0.05; **p = 0.01; ***p = 0.001.

Only low frequencies of the NK cells (1.05–5.94%) which were cultured alone were positive for CD107a (Figure 12A). Anyhow, NK cells which were co-cultured with K562 cells showed a much higher degranulation with up to 35.3% of CD107a^+^ cells (Figure 12B). In addition, the frequency of the CD107a^+^ cells upon co-cultivation was highly donor-dependent. For instance, with only 17.5% of CD107a^+^ cells donor 5 showed a much weaker degranulation than donor 3 with 35.3% of CD107a^+^ cells (Figure 12B). Statistical analysis demonstrated that degranulation of NK cells was significantly enhanced when they were cultured with K562 cells (Figure 12C).

NK cells are potent cytokine producers secreting particularly IFNγ upon recognition of target cells (Paolini et al., 2015). Release of this pro-inflammatory cytokine contributes to target cell cytolysis (Wang et al., 2012) and further strengthens immune responses, e.g., by driving Th1 differentiation, boosting macrophage function, supporting leukocyte migration to the site of infection, and enhancing major histocompatibility complex expression on infected or malignant cells (Farrar and Schreiber, 1993; Ikeda et al., 2002; Schroder et al., 2004).

**Figure 13:**
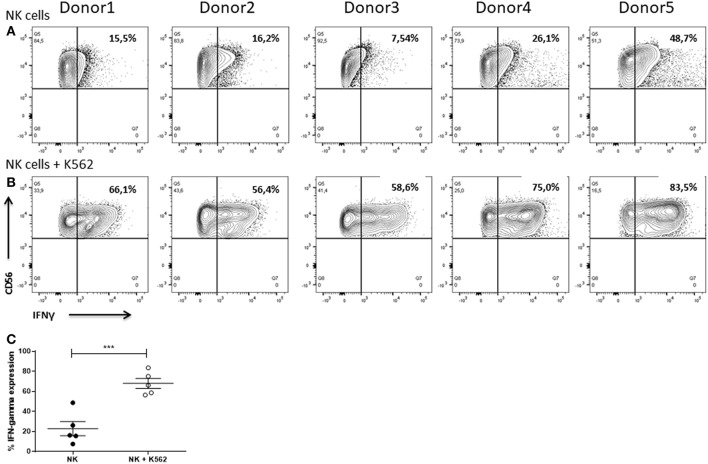
IFNγ expression of NK cells. Flow cytometric analysis of the intracellular IFNγ expression of IL-2 re-stimulated NK cells after cultivation for 6 h in **(A)** absence or **(B)** presence of K562 cells. Gates were set according to FMO controls. Graphs are representative for two independent experiments. **(C)** Values shown are the mean ± SEM. Statistical differences between both groups were determined using a paired *t*-test. *p = 0.05; **p = 0.01; ***p = 0.001.

NK cells of all donors already showed IFNγ production when cultured alone (Figure 13A). In all cases, co-cultivation of the NK cells with K562 cells for 6 h led to an increase in production of the cytokine. Nevertheless, the frequency of IFNγ^+^ NK cells differed between the donors ranging from 56.4 to 83.5% (Figure 13B). Statistical analysis demonstrated that co-cultivation with K562 cells significantly enhanced the IFNγ expression in NK cells (Figure 13C).

## Discussion

Up to now, according to the World Health Organization cancer is still the second leading cause of death worldwide and around 90% of all cancer-deaths are linked to the formation of distant metastases (Chaffer and Weinberg, 2011). Since NK cells are known to control circulating tumor cells and by this mechanism prevent metastasis formation, NK cell immunotherapy is a promising approach for cancer treatment (Brodbeck et al., 2014; Krasnova et al., 2017). In addition to their killing of circulating tumor cells, NK cells are capable of infiltrating solid tumors and a high NK cell infiltration has already been shown to correlate with a better prognosis for the patient with respect to different cancer entities (Coca et al., 1997; Gras Navarro et al., 2015; Xu et al., 2016).

After adoptive transfer of autologous NK cells showed only limited success, research focused on allogeneic NK cells as potential candidates for cancer immunotherapy (Geller and Miller, 2011). Thus, several studies have been performed in order to enhance the yields as well as the specific cytotoxicity of NK cells (Granzin et al., 2017).

In this study, we expanded human T-cell depleted PBMCs using medium supplemented with an initial activation cocktail and obtained NK cells with a high purity of more than 85% and in three cases even more than 90%. With respect to the intended therapeutic application the NK cell purity is an important factor since non-NK cells, especially T and B cells, may cause unwanted side effects including graft vs. host disease (GvHD; Ho and Soiffer, 2001; Miller et al., 2005; Ferrara et al., 2009; Skeate et al., 2013). In our process, T and B cells were barely present in the cell fractions we obtained after *ex vivo* expansion. Up to now, numerous approaches for *ex vivo* cultivation of human NK cells using a variety of different reagents including IL-2 and IL-15 as well as different culture systems have been described. In many cases, the NK cell purity stays rather low, sometimes even below 50% making our cultivation system a very attractive alternative (Childs and Berg, 2013; Granzin et al., 2015, 2017).

For full functionality and potent killing capacity NK cells require a set of different surface molecules. First, they need chemokine receptors which regulate tissue distribution and direct the cells to the tumor site. NK cells of all five donors showed expression of CXCR3, CXCR4, and CCR7 suggesting that the NK cells can be recruited to the site of action. CXCR3 expression might be of special interest with respect to cancer treatment since it regulates recruitment of NK cells into solid tumors (Wendel et al., 2008; Bernardini et al., 2016).

Apart from chemokine receptors, the NK cells also expressed all adhesion molecules required for tissue invasion and formation of the IS which is crucial for efficient target cell killing (Bryceson et al., 2005; Hoffmann et al., 2011). Most importantly, the analyzed NK cells showed expression of the NCRs NKp30, NKp44, and NKp46 as well as the activating receptors NKG2D and DNAM-1 which are all known to have a high impact on NK cell cytotoxicity (Moretta et al., 2001; Garcia-Iglesias et al., 2009; Pegram et al., 2011; Molfetta et al., 2017). Thus, many approaches already aimed at enhancing the expression of the mentioned receptors (Granzin et al., 2015). Moreover, NK cells of all five donors showed a strong expression of the Fc receptor CD16 which correlates with a high cytotoxicity of the cells (Moretta et al., 2001; Vivier et al., 2008).

As mentioned, apart from activation of the NK cells via activating receptors, they can also induce apoptosis of target cells by death ligand/death receptor interactions. In accordance with previous studies, IL-2 expanded NK cells just show a low expression of death ligands which is upregulated upon IL-2 re-stimulation. The expression of death ligands suggests that the generated NK cells are capable of inducing apoptosis of target cells. Taken together, NK cells from different donors do not seem to differ much with respect to expression of the analyzed receptors.

Results obtained from the LDH cytotoxicity assays as well as the degranulation assays demonstrated that the tested NK cells are cytotoxic and able to kill different tumor target cells even though leukemia cells were more susceptible to NK cell mediated cytotoxicity than for instance SKOV3 cells which might be explained by the epithelial morphology of the SKOV3 cells. In contrast to lymphocytes, they do usually not occur as single cells, but form firm bonds. The observed high variability between different measurements with respect to the LDH cytotoxicity assay may be explained by the heterogeneous size of the tumor cells. The measured cytotoxicity suggests that the cell adhesion molecules expressed by the NK cells managed to successfully form an IS.

Secretion of the pro-inflammatory cytokine IFNγ is an important feature of NK cells fulfilling different functions. Apart from its general immunomodulatory activity (Farrar and Schreiber, 1993; Ikeda et al., 2002; Schroder et al., 2004), IFNγ plays also an important role in tumor surveillance. It acts pro-apoptotic, inhibits tumor angiogenesis, shows anti-metastatic activity and even supports infiltration of tumors with NK cells (Smyth et al., 2002; Chawla-Sarkar et al., 2003; Wendel et al., 2008). The strong IFNγ expression by all tested NK cells argues for a high antitumor activity which could be demonstrated by the performed LDH as well as degranulation assays. Both, the cytotoxicity as well as the IFNγ expression of the NK cells seems to be influenced by the blood donor. Biological assays, such as the cytotoxicity assay used here, are generally subject to a large donor-dependent variance. The reasons for this are manifold, e.g., gender, age, nutrition, and others (Granzin et al., 2017; Pörtner et al., 2017).

These *in vitro* experiments can never totally mimic the *in vivo* situation in a given tumor. In particular, our study is not able to investigate if the expression of cell adhesion molecules correlates well with an effective recruitment of the NK cells from the blood stream to the tumor site. Furthermore, there are numerous factors including different cytokines affecting NK cell functions *in vivo*. Especially the tumor microenvironment is highly complex and may interfere with NK cell functions (Vitale et al., 2014; Gras Navarro et al., 2015). As an example, TGFβ, IL-10 or immunosuppressive enzymes may impair NK cell activity for instance by modulating the expression pattern of different receptors (Pietra et al., 2012; Castriconi et al., 2013; Stringaris et al., 2014). Therefore, we are planning to investigate the NK cell functionality in xenograft mouse models.

Taken together, a robust and GMP conform bioreactor process for expansion of NK cells with a high degree of standardization ensuring constant cell culture conditions was developed. The promising results shown here were achieved mainly by a series of innovative technical solutions, which include the technology (laminar perfusion culture), the modified bioreactor surface (additional bioreactor surface activating coating), and the optimized cultivation process (timed activation). Future work will focus on the impact of the individual measures on the performance, as this was not the intention of the studies shown here.

From our point of view, one of the most important aspects is a homogenous distribution of nutrients (particularly glucose) and gasses in the total medium flow during a cultivation run. Within the perfusion bioreactor system introduced here, homogeneity in the medium over the cultivation time is achieved by a directed laminar flow of medium forced by the meander culture bed. Medium flow in these laminar flow perfusion bioreactors is comparable to flow of blood in natural vessels, glucose concentration is regulated in a narrow range similar to *in vivo* conditions. Thus, metabolic cell stress is avoided. The steadily growing number of NK cells during expansion is compensated by an adapted ratio between circulating medium on the one hand and fresh medium feeding/cell broth removal on the other hand.

Future studies should focus on a deeper characterization of the impact of culture conditions (batch, perfusion). In general we support the view that cultivation of NK cells under static conditions, batch feeding or turbulent flow conditions (e.g., in stirred vessels) should no longer be an option for NK cell expansion as the up and down of glucose concentration and other metabolites is far away from physiological conditions in bone marrow and averts reproducible NK cell expansion. The here described *ex vivo* expansion of NK cells did not only serve the purpose to increase the number of cells, but it also generated a new NK cell type with improved antitumor potential by upregulating surface expression of distinct activating receptors (Morisaki et al., 2012; Yang et al., 2015).

Our results show that NK cells, specifically activated and expanded in an innovative, standardized perfusion bioreactor process, are highly potent and equipped with a set of different receptors mediating NK cell recruitment, adhesion as well as activation and show a high cytotoxic potential *in vitro*. For further improvement of the expansion process and an increase in cell density, the NK cells from the bioreactor 50M can be easily transferred into a single use Z®RP bioreactor 500M via a connector for sterile transportation. Several times 10^10^ NK cells with expansion factors up to 50,000 can thus be manufactured in a standardized and reproducible expansion process. Zellwerk is authorized by the local and national authorities in accordance with §13 of German Drug Act to manufacture NK cells and other immune cell therapeutics (ATMP). A clinical trial with these cells is planned.

## Ethics Statement

For this study, we obtained human blood products (whole blood) for research purposes of anonymized donors from a blood bank. As confirmed by the ethics committee in Brandenburg, use of those products does not require any positive ethics committee statement.

## Author Contributions

KB designed the experiments, conducted key studies, analyzed the data, and wrote main parts of the manuscript. US, RP, HH, ES, SL, and WD designed the study and completed the manuscript. ES, DG, and HH expanded and provided the NK cells for the experiments.

### Conflict of Interest Statement

HH is the CEO of Zellwerk GmbH and has a patent (DE: 10 2010 005 415.1:B4) as well as a patent application (EP2543719A1) for a bioreactor-based expansion process for hematopoietic cells. ES and DG work for Zellwerk GmbH. The remaining authors declare that the research was conducted in the absence of any commercial or financial relationships that could be construed as a potential conflict of interest.

## Supplementary Material

Supplementary Figure 1 Death receptor expression on tumor cells. Flow cytometric analysis of the surface expression of the death receptors **(A)** Fas, **(B)** TRAIL-1, and **(C)** TRAIL-2 on the tumor cell lines K562, PaCa5061, and SKOV3. Solid line depicts the signal for the stained tumor cells and shaded graphs show FMO controls.

Supplementary Figure 2 Cytotoxicity of NK cells toward different cancer cell lines measured by LDH cytotoxicity assay. Cytotoxicity measured by LDH cytotoxicity assay after cultivation of NK cells with **(A)** K562, **(B)** PaCa5061, and **(C)** SKOV3 cells. Cells were cultured with E:T ratios of 1:1, 2,5:1, 5:1, and 10:1. *n* ≥ 3. Values shown are the mean ± SEM.
