# Supplementary figures and images for: Mass Production of Highly Active NK Cells for Cancer Immunotherapy in a GMP Conform Perfusion Bioreactor
Source: Front Bioeng Biotechnol. 2019 Aug 13;7:194. doi: 10.3389/fbioe.2019.00194 (PMC6700243; doi:10.3389/fbioe.2019.00194)

## Slide 1
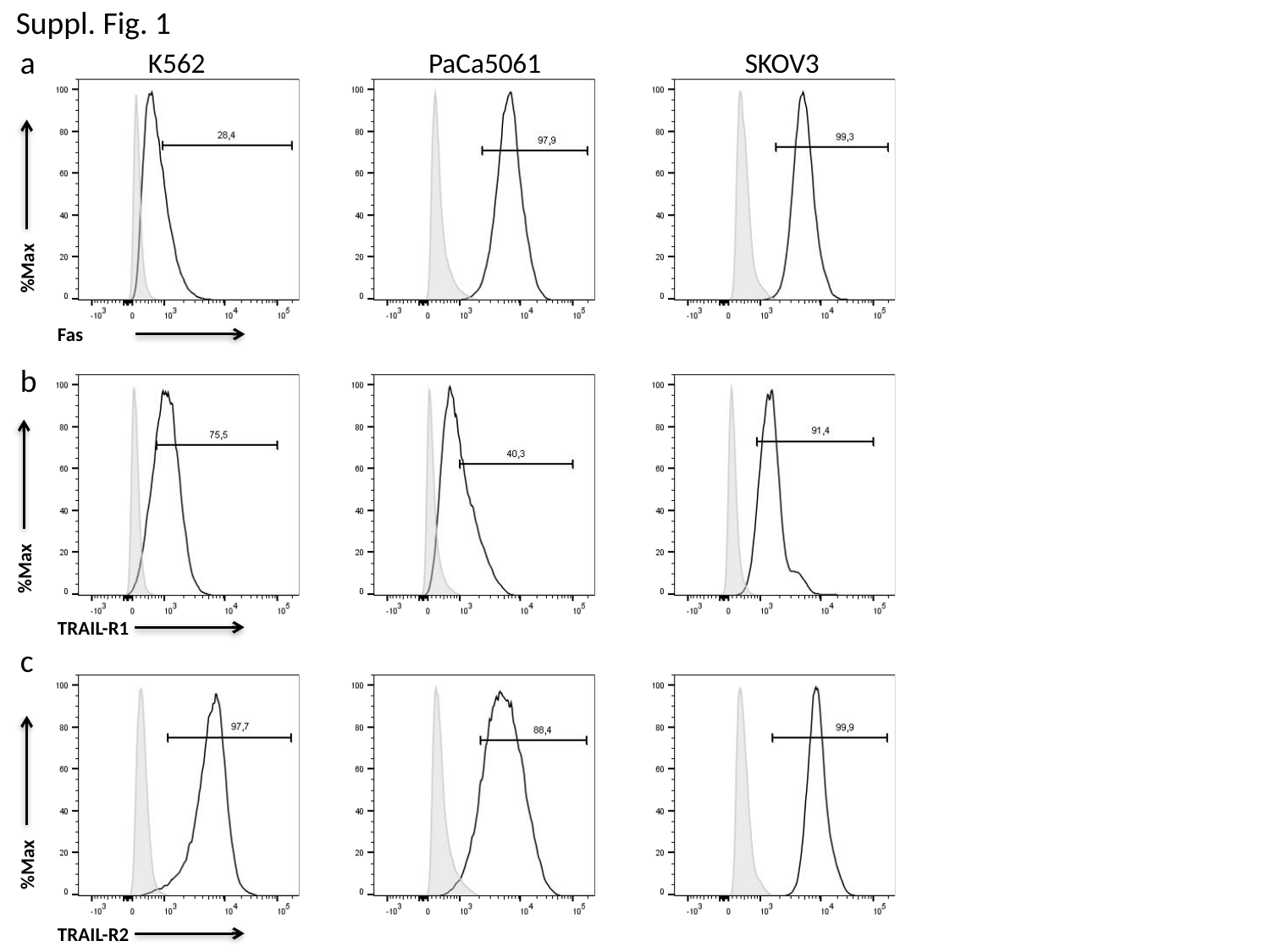

Suppl. Fig. 1
a
K562
PaCa5061
SKOV3
%Max
Fas
b
%Max
TRAIL-R1
c
%Max
TRAIL-R2

## Slide 2
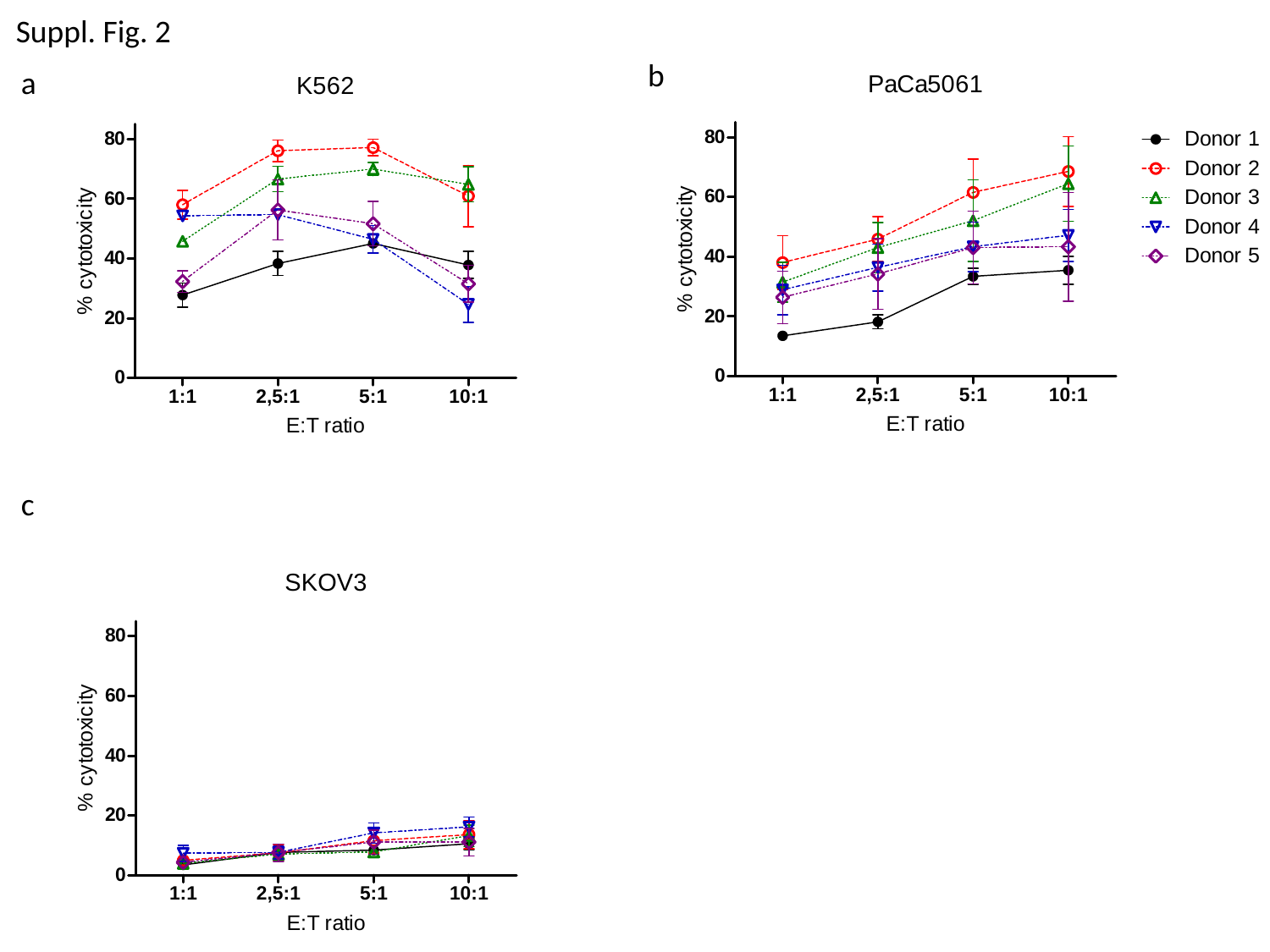

Suppl. Fig. 2
b
a
c

Supplement: Supplementary Figure 1 — Death receptor expression on tumor cells. Flow cytometric analysis of the surface expression of the death receptors (A) Fas, (B) TRAIL-1, and (C) TRAIL-2 on the tumor cell lines K562, PaCa5061, and SKOV3. Solid line depicts the signal for the stained tumor cells and shaded graphs show FMO controls. [file Presentation_1.PPTX]
